# The Sickle Cell Disease Ontology: enabling universal sickle cell-based knowledge representation

**DOI:** 10.1093/database/baz118

**Published:** 2019-11-26

**Authors:** Sickle Cell Disease Ontology Working Group, Adekunle Adekile, Kofi A Anie, Cherif Ben Hamda, Biobele Brown, Daima Bukini, Andrew Campbell, Melek Chaouch, Emile Chimusa, Catherine Chunda-Liyoka, Jemima Dennis-Antwi, Vimal K Derebail, Miriam Flor-Park, Amy Geard, Kais Ghedira, Melissa Haendel, Neil A Hanchard, Jade Hotchkiss, Mario Jonas, Muntaser Ibrahim, Clair Ingram, Baba Inusa, Adijat Ozohu Jimoh, Simon Jupp, Karen Kamga, Zainab Abimbola Kashim, Jennifer Knight-Madden, Guida Landouré, Philomene Lopez-Sall, Julie Makani, Leonard Malasa, Tshepiso Masekoameng, Gaston Mazandu, Khuthala Mnika, Nicola Mulder, Nchangwi Syntia Munung, Deogratias Munube, Liberata Mwita, Victoria Nembaware, Obiageli Nnodu, Solomon Ofori-Acquah, Kwaku Ohene-Frempong, Alex Osei-Akoto, Vivian Paintsil, Sumir Panji, Mohamed Cherif Rahimy, Charmaine Royal, Raphael Z Sangeda, Bamidele Tayo, Ines Tiouiri, Furahini Tluway, Marsha Treadwell, Leon Tshilolo, Nicole Vasilevsky, Kasadhakawo Musa Waiswa, Ambroise Wonkam

**Affiliations:** 1 Computational Biology Division, Institute of Infectious Disease and Molecular Medicine, N1.05, Werner Beit North, Faculty of Health Sciences, Anzio Road, Observatory, 7925 Cape Town, South Africa; 2 Division of Human Genetics, Department of Medicine, Faculty of Health Sciences, University of Cape Town, Anzio Road, Observatory, 7925 Cape Town, South Africa

## Abstract

Sickle cell disease (SCD) is one of the most common monogenic diseases in humans with multiple phenotypic expressions that can manifest as both acute and chronic complications. Although described more than a century ago, challenges in comprehensive disease management and collaborative research on this disease are compounded by the complex molecular and clinical phenotypes of SCD, environmental and psychosocial factors, limited therapeutic options and ambiguous terminology. This ambiguous terminology has hampered the integration and interoperability of existing SCD knowledge, and SCD research translation. The SCD Ontology (SCDO), which is a community-driven integrative and universal knowledge representation system for SCD, overcomes this issue by providing a controlled vocabulary developed by a group of experts in both SCD and ontology design. SCDO is the first and most comprehensive standardized human- and machine-readable resource that unambiguously represents terminology and concepts about SCD for researchers, patients and clinicians. It is built around the central concept ‘hemoglobinopathy’, allowing inclusion of non-SCD haemoglobinopathies, such as thalassaemias, which may interfere with or influence SCD phenotypic manifestations. This collaboratively developed ontology constitutes a comprehensive knowledge management system and standardized terminology of various SCD-related factors. The SCDO will promote interoperability of different research datasets, facilitate seamless data sharing and collaborations, including meta-analyses within the SCD community, and support the development and curation of data-basing and clinical informatics in SCD.

## Introduction

Haemoglobinopathies in general are the most common monogenic diseases of human, with sickle cell disease (SCD) the most common recessive condition (1), having variable incidence and prevalence across countries (2). SCD is mainly caused by a single-point mutation yielding a single amino acid substitution in the beta-subunit of haemoglobin, the principal oxygen transporter in red blood cells (3). Because of the protective effect of the sickle cell mutation against malaria, SCD has the highest incidence and prevalence in tropical regions, particularly in Sub-Saharan African (SSA) countries, where more than 70% of SCD patients live (4), affecting ~300 000 newborn babies every year (5). SCD affects more than 20 million people globally (6), with associated healthcare cost exceeding a billion US dollars annually (7). While it was previously thought that the SCD mutation arose independently in different regions, recent evidence has suggested a single origin of the sickle cell allele over 7000 years ago (8). The widespread increase in population migration patterns has changed the distribution of SCD frequencies in different countries, making it a global health concern (9). SCD is a chronic disease of variable phenotypes, associated with increased morbidity and mortality, and with limited effective drugs to address the clinical manifestations (10); this is particularly true in the developing world, posing a substantial burden to the healthcare system of affected countries (11). Fortunately, over the past decade, there have been numerous promising clinical trials that address all aspects of potential therapy based on sickle cell pathophysiology: from haemoglobin sickling (12), to endothelial dysfunction (13), to oxidative stress (14), to transplant (15, 16) and to gene therapy (17).

In the last decades, SCD researchers have utilized large datasets and biomedical knowledge discovery to develop a better understanding of the molecular mechanisms of the disease (18). However, a model that can incorporate results from basic biological research into clinical decision-making processes is still needed (19). Most scientific knowledge is still held in natural language text that is generally unstructured, ambiguous and subjective. With the constant evolution and growing complexity of biomedical knowledge (20), a system is needed for standardized and well-defined knowledge representation. This standard knowledge representation system should enable efficient and reliable exchange of information and integration of new knowledge by (i) more concisely defining SCD concepts, (ii) ensuring common understanding amongst scientists and clinicians and (iii) fostering more efficient data integration and interoperability with other existing systems.

Ontologies are commonly used in biomedical research to represent knowledge in a given domain, in a structured and computable format (21). An ontology defines a formal/standardized common vocabulary and relations that exist in a domain and provides an unambiguous means of communication amongst humans and between humans and computers (22). Several biomedical ontologies exist that have captured knowledge that cover some aspects of SCD, such as the Human Phenotype Ontology (HPO), which describes abnormal phenotypes encountered in human diseases (23), and the Human Disease Ontology (DO), which describes various disease domains (24). However, these ontologies do not capture all the elements that are specific to SCD, including more granular phenotypes and therapeutics that are specific to this disease state. This indicates that no ontology presently exists that can serve as a definitive and comprehensive source of SCD knowledge.

We have constructed an SCD ontology (SCDO), an integrative and universal knowledge representation system for SCD that was collaboratively developed by domain experts, including clinicians, basic scientists, bioinformaticians and data scientists. The SCDO provides a consistent vocabulary that describes key concepts and properties that establish hierarchical relationships between concepts and axioms (evidence or truths). These data are collated into a human- and machine-readable format in order to help process, reuse and reapply knowledge in biomedical research and in healthcare systems. SCDO is built around the key component, haemoglobinopathy, by linking it to phenotypes, modes of inheritance, therapeutics, diagnostics, diseases and other environmental and behavioural information for patients (personal attributes, quality of life and care). The comprehensiveness in content and structure of the SCDO makes it a model for other disease-specific ontologies.

## Methods

### Ontology development process

**Figure 1:**
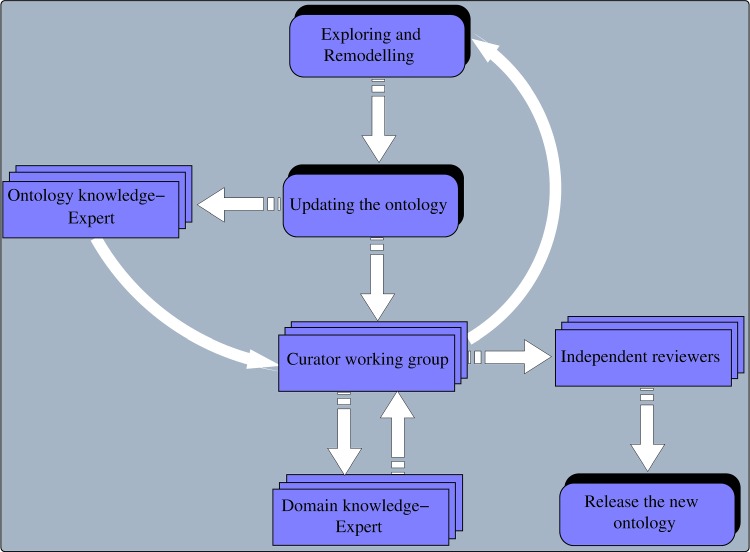
Overview of different steps in the remodelling, reviewing and release of the SCDO by curators, domain and ontology experts and independent reviewers.

The development of the SCDO was a collaborative and interactive process that included three workshops with subject matter experts from diverse backgrounds, including SCD and ontology experts, geneticists, adult and paediatric clinicians, specialists in organ systems involved in SCD, bioinformaticians and social and data scientists. At each of the annual workshops organized over 3 years, participants were split into five groups according to their expertise (phenotype, diagnostics, therapeutics, quality of life and care, disease modifiers) and each group contained 8 to 12 contributors. In between each workshop, follow-up sessions were organized in subgroups, either online or face-to-face, to continue reviewing the SCDO following steps shown in Figure 1. The work produced at each workshop was submitted to specific curation and ontology teams for further curation.

### First workshop activity and outcome

The aim of the first workshop was to (i) develop competency questions that could be addressed by the SCDO and (ii) extract the initial concepts to be included in the SCDO. The initial set of SCDO concepts was retrieved from existing ontologies and knowledge resources, including the HPO, the DO and the Online Mendelian Inheritance in Man (OMIM) database (25), as well as Genotype Ontology (GENO) at https://www.ebi.ac.uk/ols/ontologies/geno. The second set of concepts was manually extracted by SCDO curators from existing SCD guidelines and standards of care (Ghana, Nigeria, Tanzania, Jamaica, UK, Europe, Canada and USA Sickle Cell Disease Management Guidelines), as well as from literature. Different SCDO classes were merged in a spreadsheet and reviewed in two successive steps by a review team consisting of curators and SCD domain experts, including researchers and clinicians. In addition, an ontology team, which consisted of members from the European Bioinformatics Institute (EBI), Oregon State University and the SCDO team, ensured that best practices, such as OBO Foundry principles, were followed and the OWL file was produced and uploaded to BioPortal. The dynamic and iterative ontology development process is shown in Figure 1. As an outcome of this workshop, an initial list of concepts was created and further reviewed and refined by the curation team to further refine the data (26).

### Second workshop activity and outcome

After the curation team reviewed, cleaned up and fleshed out the original list of concepts to be included in the SCDO, a second workshop was convened, during which attendees reviewed the current version of the SCDO and further refined the classes and definitions. Annotation and object properties were also reviewed, and additional relationships between classes were made using the list of approved object properties. This process led to the identification of new classes that needed to be included in the ontology, probably the most notable being the ‘SCD Genotype’ and ‘SCD Causal Mutation’ classes. Discussions around the structuring of the haemoglobinopathy class and naming of haemoglobinopathies and SCD genotypes highlighted subtle differences between concepts and how ambiguous and even misleading some of the names are, thus underscoring the difficulty and importance of the SCDO’s endeavour to define these concepts.

**Figure 2:**
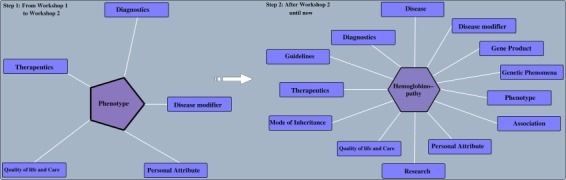
SCDO evolution before and after the second workshop. Before the ontology was built around the ‘Phenotype’ class and after, the ‘hemoglobinopathy’ class became the central class.

**Figure 3:**
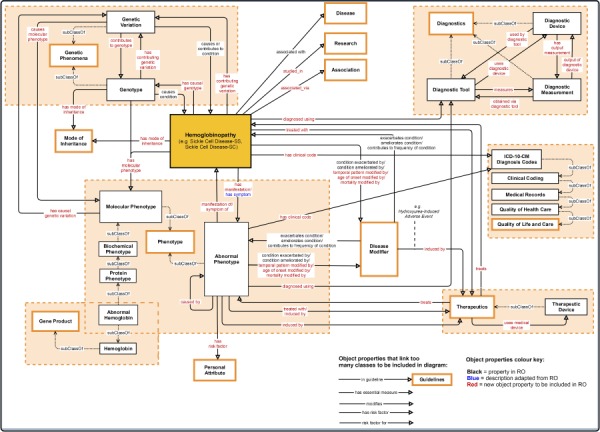
Association between the central class ‘hemoglobinopathy’ and other upper-level classes (close to the root of the ontology) in the SCDO. Properties with an asterisk also link other classes within the ontology, but these could not be shown in detail here.

### Third workshop activity and outcome

The third workshop was convened to conduct final edits on the ontology terms, with different working groups tackling the various classes. Each group had a curator who edited the terms as they were discussed. The groups were tasked with accepting the term label and text definition. Appropriate modifications were made based on the review. Some terms and relationships were added to the existing ontology; others were reclassified upon review. These classes included ‘Research’ (which includes ‘Ethnolinguistics’ as a subclass), ‘Mode of Inheritance’, ‘Association’, ‘Disease’ and ‘Gene’. Discussions after the workshop led to the ‘Gene’ class being replaced by ‘Genetic Phenomena’ and ‘Gene Product’ and to the inclusion of the upper level ‘Guidelines’ class.

### Refinement and evolution of the ontology

An ontology is always dynamic and evolving as new domain knowledge discovered is added. This results in changes within an ontology, depending on the nature of transformations applied to ontology objects. These transformations may consist of updating or removing an old object or adding a novel object, leading to ontology extension, refinement and enrichment (27). The SCDO has undergone significant enrichment, in which the central concept ‘hemoglobinopathy’, which includes SCD, has been linked to phenotypes, diagnostics, therapeutics, disease modifiers, modes of inheritance, SCD-related diseases, genetic phenomena and gene product, as well as other environmental data (personal attribute, quality of life and care for patients and research), as described in Figure 2. Properties and axioms were built mostly around this central concept in connection to other SCDO concepts, as illustrated in Figure 3.

## Results

The SCDO describes the ‘hemoglobinopathy’ class as a key aspect linking the various classes through SCDO axioms and properties as illustrated in Figure 3. This has enabled the incorporation of other haemoglobinopathies, which are related to SCD, to be included into the SCDO. These include, but are not limited to, thalassaemias and other haemoglobinopathies, which may interact or even interfere with the phenotypic manifestation of SCD.

### Current states of specific SCDO classes

**Figure 4:**
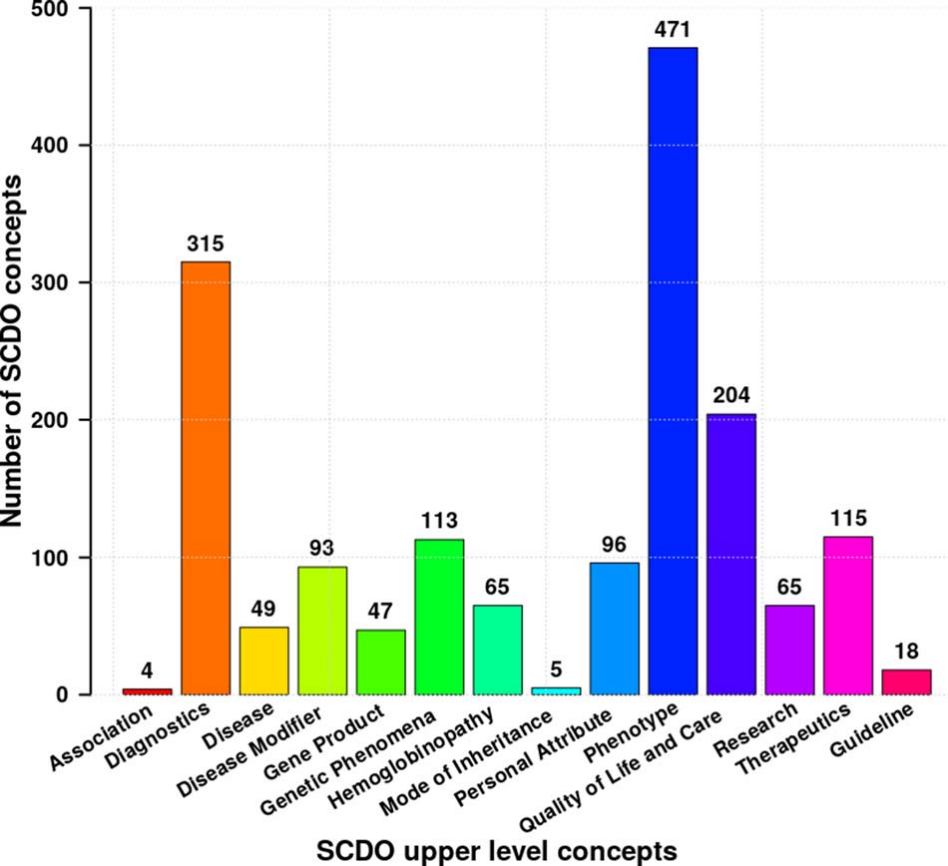
Distribution of different SCDO concepts per upper-level class with the number of associated terms in the ontology (see Supplementary Section 1 for more details about these classes).

The current SCDO has 1477 well-described terms of which 300 are terms specific to SCD and not defined previously in existing ontologies, and 1177 were retrieved terms from other ontologies (see online supplementary material for Table S1). These terms are categorized based on the upper-level classes shown in Figure 2 above, with the number of subclasses in each upper-level class shown in Figure 4. They are topologically linked by 1676 associations.

It is worth mentioning a few notable changes to some terms suggested by SCD experts. For example, the term ‘beta plus thalassemia’ has been replaced with ‘beta minus thalassemia’ based on the description of the concept ‘beta-zero’ (See Supplementary File Section 1 for more details). Table 1 provides some new terms unique to the SCDO in the phenotype class for illustration, highlighting the SCDO contribution to advancing existing knowledge and expanding scientific content in this field.

**Table 1:** Example terms specific to the SCDO phenotype upper-level class that may also be important for other ontologies, such as HPO and DO

**SCDO ID**	**SCDO term**	**Definitions or description**
SCDO:0000162	Breakthrough pain	Originally used to describe patients with cancer pain who were maintained on a stable dose of analgesics, breakthrough pain was defined as a flair-up of sudden pain unresponsive to usual therapy. Such a flare-up is usually sudden and incidental and can last from a few seconds to a few hours. There are currently no data that clearly describe or can be used to define breakthrough pain in SCD.
SCDO:0006461	Acutely severe anemia	Aplasia or haemolysis may be precipitated by another illness/infection in patients with sickle cell disease. Acutely severe anaemia is defined as Hb < 5 g/dl or a recent acute drop in HB > 2 g/dl below the individual’s steady state value. If no steady state value is available, it can be detected by the presence of acutely symptomatic anaemia (i.e. tachycardia, cardiac failure, shock).
SCDO:0007900	Embryonic hemoglobin	The type of haemoglobin present within an embryo in the first 8 weeks of gestation. Two haemoglobins, Gower 1 and Gower 2, are found in embryos of up to 8 weeks of gestation, and Haemoglobin Portland is a third normal embryonic haemoglobin found at lower levels in an embryo.
SCDO:0007161	Acute splenic sequestration crisis	Significant change in blood picture characterized by a precipitous fall in the haemoglobin level of at least 2 g/dl and accompanied by a rapidly enlarging spleen or liver (greater than 2 cm from the steady state level) and reticulocytosis above the steady state level for each individual patient. Signs of acute circulatory insufficiency, such as tachypnoea, tachycardia and hypotension, may or may not be present. It is the earliest life-threatening complication seen in patients with SCD besides pneumococcal infections.
SCDO:0006847	Aplastic crisis	An acute form of acquired red cell aplasia. A significant change in blood picture is observed, characterized by a precipitous fall in the haemoglobin level (>2 g/dl beyond steady state level) and reduced (<1%) or absent reticulocytes in the peripheral blood. The total white blood cell or platelet counts may or may not be affected. In addition, there is no significant increase in the unconjugated fraction of serum bilirubin.
SCDO:0009664	Abdominal vaso-occlusive crisis	Abdominal distension with generalized abdominal tenderness (no rebound tenderness) and reduced bowel sounds. Abdomen moves with respiration. Vomiting/diarrhoea is not common. It is thought to occur secondary to the occlusion of mesenteric vessels.
SCDO:0000812	Non-specific acute lower respiratory tract episode	Includes acute respiratory episodes with lower respiratory tract signs that do not meet the criteria for other diagnoses. May include episodes that would have been diagnosed as ACS were radiographic facilities available.
SCDO:0008625	Right upper quadrant syndrome	Characterized by pain and discomfort in the right upper quadrant (RUQ) of the abdomen caused by a number of possible aetiologies in sickle cell disease. Causes of RUQ pain may be divided into pain originating from the liver or gall bladder versus other origins of abdominal pain in that region.
SCDO:0006909	Hyperhemolytic Crisis	Significant change in blood picture characterized by a precipitous fall in the haemoglobin level associated with jaundice, marked reticulocytosis and polychromasia on the blood smear, increased unconjugated hyperbilirubinemia and increased urobilinogen content in urine above the steady state level for each individual patient.
SCDO:0002039	Zinc deficiency	A deficiency of the essential metal zinc; an essential cofactor for many enzymes. Zinc deficiency is caused by a lack of zinc in the diet, loss of zinc after absorption, for example through loss through burns, inability to absorb zinc or increased loss through exercise.
SCDO:0008623	Vaso-occlusion	The obstruction of blood vessels by altered erythrocytes that can result in pain, anaemia and tissue ischemia.
SCDO:0004888	Functional hyposplenism	An acquired disorder caused by several haematological and immunological diseases and characterized by impairment of splenic function.
SCDO:0006835	Normal hemoglobin	Haemoglobins that present no inherited health condition phenotype susceptible to undergo alterations in the red blood cells.
SCDO:0004187	Hemolytic crisis	May be caused by an acute VOC, malarial infection or oxidant drug exposure in individuals with concomitant glucose-6-phosphate dehydrogenase (G6PD) deficiency. Haemolytic crisis may be distinguished from aplastic crisis by the finding of a reticulocytosis as opposed to a reticulocytopenia.
SCDO:0001229	Vaso-occlusive crisis	Pain resulting from tissue ischemia as a result of blockage of blood vessels, occurring in a variety of vascular beds, but most commonly in the bone or bone marrow and requiring analgesic medication.
SCDO:0004576	Chronic hypersplenism	Chronic splenic sequestration associated with enlarged spleen and cytopenia with anaemia and reduction in white blood cells and platelets. The anaemia is usually chronic in nature and patients rarely present with signs of heart failure.
SCDO:0000225	Chronic complications of sickle cell disease	A condition that co-exists or follows from sickle cell disease and that has a slow, creeping onset, slow progress and long continuance of disease manifestations.
SCDO:0000895	Phenotype of sickle cell disease	A (combination of) quality(ies) of some or all sickle cell disease individuals, determined by the interaction of the genetic make-up of these individuals (with regard to sickle cell disease) and their environment.
SCDO:0001135	Acute sickle cell crisis	Refers to a worsening, over a short period of time, of the symptoms and signs of SCD; usually associated with pain and/or shortage of blood (anaemia). Can be suspected in a person with sickle cell disease who presents with a sudden onset of pain, infection, anaemia or other symptoms such as stroke or priapism. Acute pain frequently occurs spontaneously, but may be precipitated by infections, skin cooling, dehydration or stress.
SCDO:0001023	SCD related pain	Pain resulting from the presence of sickle cell disease (SCD). Such pain can be acute, chronic or a mixture of the two.
SCDO:0000233	Chronic sickle cell pain	Pain that does not resolve and lasts for more than 3 months.
SCDO:0001604	Steady state	This is a period when the patient with sickle cell anaemia is free of infection, pain or other disease processes.
SCDO:1000061	Altered level of normal hemoglobin present in SCD	An altered level of normal haemoglobin (Haemoglobin A (Hb A), Haemoglobin A2 (Hb A2) or Haemoglobin F (Hb F, fatal haemoglobin) in the blood, which may be seen in those suffering with sickle cell disease (SCD).
SCDO:0000007	Abnormal hemoglobin structure present in SCD	A structurally abnormal haemoglobin that occurs in one or more forms of sickle cell disease (SCD).

### Evaluation of the ontology

Generally, data-driven quality evaluation of a given knowledge-based system, such as an ontology, is based on its performance in associated applications, for which there is a need for an independent specification against which the ontology should be assessed. However, since SCDO is only in its infancy, instead, we used rules and questions sketched by SCD experts, referred to as competency questions, to check whether the ontology addresses its scope by ensuring that it contains appropriate information to satisfy these rules or answer these questions.

**Figure 5:**
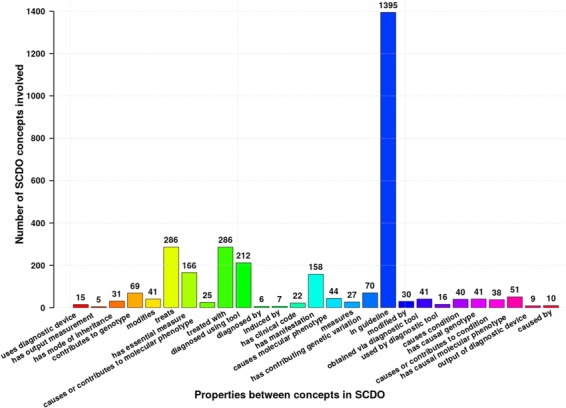
Different properties and axioms defined between different SCDO concepts to satisfy rules set by SCD experts or to answer competency questions. Numbers at the top of bars represent the occurrence frequency of the association in the ontology. ^*^Authors: Sickle Cell Disease Ontology Working Group.

Competency questions (see Supplementary File Section 4) were related to SCD signs, symptoms and complications, focusing specifically on pain, skin, chest infection, stroke and kidney disease to explore potential connections for knowledge discovery. To enable SCDO to answer these questions and its structure to contain different rules and constraints, we have modelled SCDO in such a way that it contains concepts specific to SCD (see Table 1), appropriate relations and properties or axioms as shown in Figure 5 and illustrated in the SCDO schema (Figure 3). We expect that SCDO can serve as a template for knowledge acquisition and reuse and is applicable to future SCD research applications, specifying a standardized common vocabulary verified by SCD experts from diverse specialized backgrounds.

### SCDO release and licence

SCDO is released every 2 months with possible special releases when there are significant incidental changes. It is freely available under the Creative Commons Attribution 4.0 Unported License (CC:https://creativecommons.org/licenses/by/4.0/legalcode) and further copyrighted to maintain the quality and integrity of the vocabularies, meaning that any modification to the SCDO can only be done by SCDO developers and curators.

### Accessing the ontology

A website was built to serve the SCDO at http://scdontology.h3abionet.org, hosted on servers at the Pasteur Institute in Tunis (http://tesla.pasteur.tn). To facilitate the viewing and searching of terms, the Ontology Lookup Service (OLS) auto-complete widget was installed, and its associated search bar was placed on the main page. A global comment system using Disqus was integrated under each term’s web page, to enable discussion and to connect conversations across the website. Search and viewing mechanisms were also integrated in the EBI OLS, which is directly accessible via https://www.ebi.ac.uk/ols/ontologies/scdo. The current OWL and OBO files are accessible via the GitHub repository (https://github.com/scdodev/scdo-ontology).

## Discussion

SCDO is the first and most comprehensive standardized human- and machine-readable resource that unambiguously represents terminology and concepts about SCD and other haemoglobinopathies for researchers, patients and clinicians. The SCDO was derived through an exhaustive, iterative and collaborative process drawing on expertise of individuals across multiple disciplines worldwide. Prior to its development, existing ontologies included items relevant to SCD, but none of these ontologies fully encompassed all elements of this uniquely complex and frequent genetic disease. The utilization of the SCDO can have far-reaching outcomes in areas where the burden of disease is greatest, narrowing the gap of evidence-based SCD care management.

### SCDO and other disease ontologies

Disease ontologies provide coverage of disease and disorder domains and help in disease annotations in different biomedical applications, supporting clinical and research applications, including clinical data aggregation of electronic health records, clinical decision processes and literature-based mining. In most cases, existing disease-related ontologies (http://www.obofoundry.org/) attempt to describe global disease domains and abnormal phenotypes encountered in disease conditions, including the DO (24), the HPO (23), the Mouse Pathology Ontology (MPATH—http://www.pathbase.net/), the Mammalian Phenotype Ontology (MPO—http://obofoundry.org/ontology/mp.html) (26) and Phenotype And Trait Ontology (PATO—http://obofoundry.org/ontology/pato). As pointed out previously, these ontologies do not capture concepts specific or unique to SCD. The SCDO team is submitting terms and properties for inclusion in existing ontologies including the HPO, the OBO Relations Ontology (RO) (27) and the Data Usage Ontology (DUO) (http://www.ontobee.org/ontology/DUO).

There are specialized and disease-specific ontology projects, such as the Infectious Disease Ontology (IDO—http://www.infectiousdiseaseontology.org/), which aims at providing a set of interoperable ontologies that should cover entities related to specific pathogens and diseases, including human immunodeficiency virus (HIV), dengue fever, influenza, malaria and tuberculosis. Another hematology-related ontology project is the Blood ontology (BLO—http://mba.eci.ufmg.br/BLO/, http://bioportal.bioontology.org/projects/Blood_Ontology), integrating different existing blood terminologies. More recently, an ontology of Type 2 diabetes (http://purl.bioontology.org/ontology/DIAB), DIAB, has been developed around Phenotype (28) as have other existing disease-specific ontologies. However, there is neither deterministic nor systematic mapping between phenotype (manifestation) and disease (condition), except for some specific ‘pathognomonic’ manifestations. Thus, for consistency, SCDO is built around the ‘hemoglobinopathy’ concept, which is then linked to other disease-related aspects, such as diagnostics, therapeutics and phenotypes, as well as several other aspects pertinent to SCD research, patient outcomes and care, including Personal Attribute with Ethnolinguistic groups, Quality of Life and Care, Guidelines and Disease Modifier, which may orient phenotypic manifestations and inform clinical management. This suggests that the SCDO development approach may constitute a model that can drive implementation of other disease-specific ontologies.

### Challenges encountered in the development

The main aim is to provide an ontology that will conceptualize SCD disease management and research domains and be effectively applicable across a range of biomedical applications (29). Designing such an ontology is a tedious and daunting process, requiring expertise from varying specialized backgrounds, including geneticists, adult and paediatric clinicians, specialists in organ systems involved in SCD, biologists, philosophers, anthropologists, ontologists and data scientists. These experts should share a common understanding of existing SCD knowledge, make domain assumptions explicit, discuss the scope of the model through competency questions and define SCD concepts, relations and other axioms to be included (30). However, it was not always easy to converge to a single conceptualization of a domain, and this process was particularly time-consuming as each expert conceptualizes the domain depending on how he/she came to understand it, which is often related to particular experiences.

It is known that an ontology is never complete and always dynamic, and we expect the SCDO to continue to evolve for many years considering the context in which it has been developed. Currently, the SickleInAfrica consortium (https://www.sickleinafrica.org), which includes the Sickle Pan-African Research Consortium (SPARCo), Sickle Africa Data Coordinating Center (SADaCC) and Sickle Pan African Network (SPAN), intends to collaboratively collect large-scale SCD patient datasets, using the SCDO to harmonize these datasets and facilitate data integration, information retrieval and analysis. SCDO should be able to handle the challenges of a potential exponential growth of SCD datasets by keeping SCD knowledge updated, possibly on a daily basis, revealing gaps in the existing knowledge and identifying new hypotheses and research questions. To achieve this, some of the processes involved in ontology update and evolution need to be automated to minimize time and resources invested by curators.

### Limitations

Despite the successful international collaborative effort to develop this SCDO, there were some notable limitations. The absence of experts from South Asia (i.e. India) leaves a void of characteristics that might be specific to that region, which has one of the highest prevalence rates of SCD in the world (11). Furthermore, the SCDO in its current iteration is available only in the English language, limiting its applicability to regions where the majority of individuals are non-English speakers. While constructing SCDO has been a successful effort, it has yet to be applied; hence, the full strength of its utility is unknown. Some efforts are in progress to mitigate these limitations.

### Future directions

Future efforts include translation of the ontology into French and Portuguese, to make it more accessible to native French and Portuguese speakers in Africa and beyond. In addition, we aim to create a lay person version of the SCDO to make terms accessible to non-medical experts, as has been previously done with the HPO (31). Offering an SCDO layperson-friendly version enables the use of SCDO in patient data collection forms, allowing patients to perform standardized self-phenotyping.

To enable semantic interoperability with other ontologies, future efforts will be made to add logical definitions or equivalence axioms that utilize other OBO Foundry ontologies (32). This will allow for machine readable definitions and allow automated reasoning and inferencing. Moreover, the annotations of SCDO terms will continue to be enriched in numerous ways to ensure a high coverage of existing and future SCD knowledge. An example would be to include terms for measurement units into the ontology and to link diagnostic measurements to their relevant units and information about the measurement normal ranges, amongst many other aspects that will enrich SCDO terms.

An SCDO application is already under way. Using the SCDO design, an SCD-based case report form (SCD-CRF) has been developed. The SCD-CRF provides a standardized tool that can be utilized and adapted as needed in multi-national cohort studies. Because it is linked to and derived from the SCDO, studies utilizing the SCD-CRF will capture data more uniformly in comparison to prior studies for which tools were created *ad hoc* for a single study. Currently, research in rare disease has been hampered by a lack of standardization across studies with individual research groups employing definitions for exposures, measures and outcomes that may differ substantially. Developing a knowledge base and data capture tools for a specific disease will impose greater rigor on these cohort studies as well as better inter-study comparability if standardized, agreed-upon definitions are used. The process herein described for the SCDO offers a model approach for the construction of other genetic disease-specific ontologies.

## Conclusion

The SCDO was created via a collaborative and iterative process, and this first SCDO release provides a comprehensive description of clinically relevant aspects of SCD, standardizing common SCD vocabulary. This will facilitate seamless data sharing and collaborations including meta-analysis within the SCD community and support the development and curation of data-basing and clinical informatics in SCD. The ontology will continue to be developed by the SCD community using best practices and guidelines. We hope that the SCDO will prove to be a valuable resource for researchers, clinicians, patients and anyone affected by SCD and facilitate the global unification of SCD knowledge. Currently, the SCDO represents the most comprehensive compendium in the SCD field to our knowledge. We anticipate that the SCDO will drive the expansion of scientific content in this field and enhance information structuring, searching and retrieval and can lead to new hypotheses and discoveries.

## Authors’ contributions

1. Project leads: N.M., A.W.
2. Project coordinator: V.N.
3. SCDO key curators: J.H., A.G., K.M.
4. SCDO Ontologists and Developers: G.K.M., S.J., K.G., C.B.H., M.H.
5. SCDO Content Providers: K.O.-F., all working group chairs and members
6. SCDO Working Group Chairs: K.A., A.C., F.C., C.C.-L., N.H., J.K.-M., D.M., O.N., B.T., M.T., C.R., S.O.-A., K.O.-F.

## Supplementary Material

Mulder_Wonkam_SCD_supplementary_file_revised_baz118

## Notes

**Availability:** Ontology URL https://bioportal.bioontology.org/ontologies/SCDO.

**Contact:** nicola.mulder@uct.ac.za, ambroise.wonkam@uct.ac.za

## Sickle Cell Disease Ontology Working Group in alphabetical order

Adekunle Adekile^1^, Kofi A. Anie^2^, Cherif Ben Hamda^3^, Biobele Brown^4^, Daima Bukini^5^, Andrew Campbell^6^, Melek Chaouch^3^, Emile Chimusa^7^, Catherine Chunda-Liyoka^8^, Jemima Dennis-Antwi^9^, Vimal K. Derebail^10^, Miriam Flor-Park^11^, Amy Geard^12,13^, Kais Ghedira^3^, Melissa Haendel^14^, Neil A. Hanchard^15^, Jade Hotchkiss^7^, Mario Jonas^7^, Muntaser Ibrahim^16^, Clair Ingram^7^, Baba Inusa^17^, Adijat Ozohu Jimoh^18^, Simon Jupp^19^, Karen Kamga^7,20^, Zainab Abimbola Kashim^18^, Jennifer Knight-Madden^21^, Guida Landouré^22,23^, Philomene Lopez-Sall^24^, Julie Makani^5^, Leonard Malasa^5^, Tshepiso Masekoameng^7^, Gaston Mazandu^7^, Khuthala Mnika^7^, Nicola Mulder^25^, Nchangwi Syntia Munung^26^, Deogratias Munube^27^, Liberata Mwita^5^, Victoria Nembaware^7^, Obiageli Nnodu^28^, Solomon Ofori-Acquah^29,30^, Kwaku Ohene-Frempong^31^, Alex Osei-Akoto^32^, Vivian Paintsil^33^, Sumir Panji^25^, Mohamed Cherif Rahimy^34^, Charmaine Royal^35^, Raphael Z Sangeda^36^, Bamidele Tayo^37^, Ines Tiouiri^3^, Furahini Tluway^5^, Marsha Treadwell^38^, Leon Tshilolo^39^, Nicole Vasilevsky^40^, Kasadhakawo Musa Waiswa^41^, Ambroise Wonkam^7^

1. Kuwait University, Kuwait City, Kuwait.

2. Haematology and Sickle Cell Centre, London North West University Healthcare NHS Trust & Imperial College London, London, United Kingdom

3. Laboratory of Bioinformatics, Biomathematics and Biostatistics (LR16IPT09), Institute Pasteur of Tunis, University of Tunis El Manar, Tunis, Tunisia

4. Department of Paediatrics, College of Medicine, University of Ibadan, Ibadan, Nigeria

5. Sickle Cell Programme, Department of Haematology and Blood Transfusion, Muhimbili University of Health and Allied Sciences, Tanzania

6. Division of Hematology, Children’s National Medical Center, George Washington School of Medicine and Health Sciences, Washington, DC, USA

7. Division of Human Genetics, Department of Pathology, Faculty of Health Sciences, University of Cape Town, Cape Town, South Africa

8. University Teaching Hospitals - Children’s Hospital, University of Zambia, Lusaka, Zambia

9. Sickle Cell Foundation of Ghana. East Legon-Accra, Ghana

10. UNC Kidney Center, Division of Nephrology and Hypertension, University of North Carolina at Chapel Hill

11. Onco Hematology Unit, Instituto da Criança, Hospital das Clínicas, Universidade de São Paulo, Brazil

12. Gene Transfer Technology Group, UCL EGA Institute for Women’s Health, University College London, London, UK

13. University of the Witwatersrand, School of Pathology, Antiviral Gene Therapy Research Unit, Health Sciences Faculty, South Africa

14. Department of Environmental and Molecular Toxicology, Oregon State University, Corvallis, OR, USA

15. Department of Molecular and Human Genetics, Baylor College of Medicine, Houston, TX, USA

16. Institute of Endemic Diseases, - University of Khartoum, Sudan

17. Department of Paediatric Haematology, Evelina Children’s Hospital, Guy’s and St Thomas NHS Trust, London

18. Department of Genetics, Genomics and Bioinformatics - National Biotechnology Development Agency (NABDA), Abuja, Nigeria

19. European Molecular Biology Laboratory, European Bioinformatics Institute (EMBL-EBI), Wellcome Trust Genome Campus, Hinxton, United Kingdom

20. FMBS, University of Yaounde

21. The Sickle Cell Unit, Caribbean Institute for Health Research, the University of the West Indies

22. Faculté de Médecine et d’Odontostomatologie, USTTB, Mali

23. Service de Neurologie, CHU du Point “G”, Bamako, Mali

24. Department of Pharmacy, Biochemistry Unit, Cheikh Anta Diop University, Dakar, Senegal.

25. Computational Biology Division, IDM, CIDRI-Africa Wellcome Trust Centre, University of Cape Town Faculty of Health Sciences Anzio Road, Observatory, Cape Town, South Africa,

26. Department of Medicine, University of Cape Town, South Africa

27. Department of Paediatrics and Child Health, Makerere University/Mulago National Referral Hospital, Kampala, Uganda

28. Department of Haematology & Blood Transfusion, College of Health Sciences, University of Abuja, Abuja, Nigeria

29. Dean, School of Biomedical and Allied Health Sciences

30. Director, SickleGenAfrica:Sickle Cell Disease Genomics Network of Africa

31. President, Sickle Cell Foundation of Ghana

32. Dept. of Child Health, School of Medicine and Dentistry, Kwame Nkrumah University of Science and Technology/Komfo Anokye Teaching Hospital, Kumasi, Ghana

33. Komfo Anokye Teaching Hospital, Kumasi, Ghana

34. Centre de Prise en Charge Medicale Integree du Nourrisson et de la Femme Enceinte atteints de Drepanocytose, (The National Sickle Cell Disease Institute), Cotonou, Republic of Benin

35. Departments of African & African American Studies, Biology, and Community & Family Medicine, Duke University, Durham, USA

36. Department of Pharmaceutical Microbiology, Muhimbili University of Health and Allied Sciences, Tanzania

37. Department of Public Health Sciences, Loyola University Chicago Stritch School of Medicine, Maywood, IL, USA

38. UCSF Benioff Children’s Hospital Oakland, Department of Hematology/Oncology 747 52nd Street Oakland, CA USA 94609

39. Centre de Formation et d’Appui sanitaire/MONKOLE, Kinshasa, DR Congo

40. Oregon Clinical & Translational Research Institute, Department of Medical Informatics and Clinical Epidemiology, Oregon Health & Science University, Portland, USA

41. Department of Medicine, Makerere University College of Health sciences, University college of Health sciences
