# Supplementary material for: The Sickle Cell Disease Ontology: enabling universal sickle cell-based knowledge representation
Source: Database (Oxford). 2019 Nov 26;2019:baz118. doi: 10.1093/database/baz118 (PMC6878945; doi:10.1093/database/baz118)
Supplement: Mulder_Wonkam_SCD_supplementary_file_revised_baz118 [file mulder_wonkam_scd_supplementary_file_revised_baz118.docx]

**Supplementary File**

**The Sickle Cell Disease Ontology: Enabling universal sickle cell-based knowledge representation**

**Section 1: Describing sickle cell disease ontology (SCDO) high level classes**

***1. Hemoglobinopathy:*** A hemoglobinopathy is an inherited disorder of the structure and/or synthesis of hemoglobin, with a variable expression ranging from benign to severe. This class was initially a sub-class of “Phenotype”, but was subsequently moved to a more prominent position in the ontology as one of the upper-level classes. Initially it contained two main sub-classes, namely “Sickle Cell Disease” (containing “Sickle Cell Disease Variant” and “Sickle Cell Disease-SS”) and “Hemoglobinopathy Occuring in Heterozygous State in Sickle Cell Disease Variants”. The classification of hemoglobinopathies was then reworked a few times with the outcome being five main classes, namely “Quantitative Hemoglobinopathy”, “Structural Hemoglobinopathy”, “Structural and Quantitative Hemoglobinopathy”, “Sickle Cell Disease” (containing “Variant Sickle Cell Syndrome”, “Sickle Cell Disease-SS” and “SCA Plus Alpha Thalassemia”) and “Hemoglobin Trait”. This class was developed to be central for SCDO and contains 65 classes, including what aims to be a comprehensive categorisation of different forms of the sickle cell disease (SCD), within the “Sickle Cell Disease” class.

A few notable changes to some terminology have been introduced under the direction of SCD experts. Most notably, the term “Beta Plus Thalassemia” has been replaced with “Beta Minus Thalassemia”, and all terms involving this form of beta thalassemia have been likewise changed to contain “minus” instead of “plus”. Experts were of the opinion that “beta-plus” is a misnomer because “beta-zero” implies zero globin production and as has been the convention. “Beta-plus” results in reduced, not increased, beta-globin production, therefore logically the correct designation should be “beta-minus” when there is reduced globin production. The consensus was that inheritance of more than two beta-globin genes or increased production of beta globin would be correctly designated as “beta-plus”. Likewise, for mutations that result in multiplication of the beta globin gene, “beta-plus” would be an appropriate designation.

***2. Phenotype:*** Defined as an observable feature of an organism resulting from the interaction of its genetic make-up and environment, differentiating specific instances or individuals of a species from other instances of the same species. This SCDO upper level class is not only limited to disease specific disorders, but also includes other disease entities which may be modified by the presence of sickle cell disease. A total of 471 classes were created and reviewed. Even though the SCDO Phenotype class was modelled using existing ontologies, especially HPO, it contains concepts specific to SCD defined by experts with individual clinical characteristics affecting SCD patients and agreed on by the curation team. These terms are not found in the existing ontologies and **Table 1** provides some of them for illustration. They are extendable to other abnormal phenotypes and can be included in other ontologies. This illustrates the SCDO contribution to advancing existing knowledge and expanding scientific content in this field.

***3. Diagnostics:*** Diagnostics are defined as diagnostic tools (including instruments, laboratory assays and methods of patient examination) that are used for differential diagnosis, the measurements obtained using these tools, and the diagnostic devices used in obtaining the measurements. 315 Diagnostics terms were reviewed, and most of the terms and their descriptions were accepted. Two diagnostic terms were labelled as redundant and as such were recommended to be removed. The term “Histologic Smear” was not a terminology used in clinical practice so this was changed to “Diagnostic Histology Test”. “Health Insurance Coverage” was recommended to be moved to the Quality of Life and Care concept. For the term ‘Prenatal Screening for Sickle Cell’, the definition was revised to remove language that could have moral, ethical and legal implications in most African settings.

***4. Quality of Life and Care:*** This is defined as the subjective measurement of an individual's sense of well-being and ability to enjoy life, and the levels of excellence which characterize the health service or health care provided to individuals based on accepted standards of quality. The 233 classes in this branch were reviewed by a working group, which later divided into two sub-groups that worked separately to define the terms and synonyms under ‘quality of life’ and ‘quality of care’, respectively with references. These two sub-groups subsequently came together to agree on the definitions of the terms. Some of the quality of life and care terminology relating to treatment was transferred to the ‘Therapeutics’ category where appropriate and finally this upper level concept contains 204 terms.

***5. Disease Modifier:*** Defined as factors that may induce substantial clinical variability so that patients with the same conditions may develop a very severe form of the disease, a mild form or show no symptoms at all. Examples of modifiers included environmental factors, age, modifier genes, allelic variation and other co-morbid diseases. This branch contained 191 classes. Upon review of this branch, genetic modifiers were flagged to be reclassified as a subclass of hemoglobinopathy as these were felt to define rather than modify the underlying disease. Other definitions were also refined to include details of the variants and amino acid changes. Furthermore, the Gene Product and Genetic Phenomena concepts containing 47 and 113 sub-classes, respectively, were removed from the 'disease modifier' class, which now contains 93 sub-classes.

***6. Research:*** Defined broadly as the critical and exhaustive investigation or experimentation, having as its aim the discovery of new facts and their correct interpretation, the revision of accepted conclusions, theories, or laws in the light of newly discovered facts, or the practical application of such new or revised conclusions, theories, or laws. This includes ethical considerations in research. The working group adopted 65 key terms in this class that encompass different research aspects; study design; ethical principles such as informed consent, autonomy, confidentiality and beneficence; and descriptions of data and sample handling including database, biobank and material transfer agreement.

***7. Therapeutics:*** Defined as procedures concerned with the comprehensive treatment or prevention of diseases including those directed to the specific disease entity and its co-morbidities. The working group reviewed 115 terms for clarity, adherence to standardized guidelines and clinical practice. Those terms deemed not acceptable were redefined, and the sources for the new definition added. For new definitions, we utilized MESH subject headings, WebProtege and standard published literature. Some terms were felt to be misclassified as therapeutics and were redirected to the appropriate class after discussion with the larger working group. Eighteen terms were felt to be better suited in the diagnostic category, two terms in quality of life and one term in the phenotype group. The term “Right Heart Catheterization” belonged to both therapeutics and diagnostics classes. New definitions were added for the following terms: penicillin, vitrectomy, adjuvant, genetic service, antifungal, acute sickle cell management, echocardiogram and chronic sickle cell pain. Based on the recent approval of L-glutamine in the treatment of sickle cell disease by the United States Government, Federal & Drug Administration in 2017 (<https://www.fda.gov/Drugs/InformationOnDrugs/ApprovedDrugs/ucm566097.htm>), this term and its definition were added.

***8. Personal Attribute:*** Defined as distinguishing qualities or prominent aspects of an individual person, including subclasses like demographic information, such as age or birthplace, and medical history. This branch contained 96 subclasses. Upon review, some classes were reclassified as diagnostic features or research concepts (for example, longitudinal studies) and, currently, this class contains 88 concepts.

Finally, other related classes include disease, mode of inheritance, guidelines, associations, gene product and genetic phenomena containing 49, 5, 18, 4, 47 and 113 terms, respectively.

As pointed out in the main manuscript, SCDO contains terms specific to SCD and those retrieved or adapted from other ontologies and databases. The number of classes specific to SCDO vs reused classes has now are shown in Table S1 and the “existence in other ontologies” annotation property was used to ascribe an “Existence Status” to each term, which describes the existence of the term in other ontologies.

**Table S1:** Frequency summary of classes specific to SCDO vs reused classes.

| **Existence Status** | **Explanation of status** | **No. terms** | **% terms** |
| --- | --- | --- | --- |
| **Sufficient** | Exists in other ontology and has appropriate description | 831 | 56.6 |
| **Suggest update to description** | Used term from existing ontology but will suggest they update their description to ours | 81 | 5.5 |
| **Suggest update to label** | Used term from existing ontology but will suggest they update their label to ours | 51 | 3.5 |
| **Suggest update to label and description** | Used term from existing ontology but will suggest they update their label and description to ours | 5 | 0.3 |
| **Few but definitions not available** | Term exists in few ontologies but has not been given a description in any | 1 | 0.1 |
| **Few but definitions not freely available** | Term exists in few ontologies but the description is not freely available | 99 | 6.7 |
| **Few but definitions not specific enough** | Term exists in few ontologies but the definitions are not specific enough for the SCDO’s needs | 26 | 1.8 |
| **Not relevant to context of sickle cell** | Term exists in other ontologies but the definitions are not relevant to the SCD field | 21 | 1.4 |
| **Negligible** | No description or outdated ontology | 52 | 3.5 |
| **None** | Not in any existing ontology | 300 | 20.4 |

**Section 2: Relations Ontology**

We tried to exhaustively include concepts relevant to SCD and to identify attributes associated with different concepts, where possible. In the process, we re-used some object and annotation properties from existing ontologies but also needed to create new properties when we did not find relevant ones in existing ontologies. Our list of 37 new object properties to be requested for inclusion in the Relations Ontology (RO) is shown in **Table S2**.

**Table S2:** List of new object properties required by the SCDO to be included in the Relations Ontology (RO)

| **SCDO ID** | **Property Name** | **Description** | **Inverse Properties** | **Suggested Super property** |
| --- | --- | --- | --- | --- |
| SCDO:0000474 | has output measurement | A relation between a device (diagnostic device) and the recording of the output (diagnostic measurement) of the device. |  | has output |
| SCDO:1000194 | measures | A relation between a diagnostic tool and the diagnostic measurement that it measures. |  |  |
| SCDO:1000190 | obtained via diagnostic tool | A relationship between a diagnostic measurement and the diagnostic tool used to produce the measurement. |  |  |
| SCDO:1000191 | used by diagnostic tool | A relationship between a diagnostic device and the diagnostic tool by which the device is used. |  |  |
| SCDO:1000192 | uses diagnostic device | A relation between a diagnostic tool (test, method, assay, etc.) and a diagnostic device, where the diagnostic device is used in the application of the diagnostic tool. |  |  |
| SCDO:1000193 | output by diagnostic device | A relation between a diagnostic measurement and a diagnostic device, where the diagnostic measurement is the output of the diagnostic device. |  | has output |
| SCDO:0000663 | diagnosed using tool | A relation between a phenotype that is a diagnosis and a diagnostic tool used in making the diagnosis. |  |  |
| SCDO:1000220 | diagnosed using measurement | A relation between a phenotype that is a diagnosis and a diagnostic measurement used in making the diagnosis. |  |  |
| SCDO:1000219 | diagnosed by | A relation between a condition that is a diagnosis and a phenotype used in making the diagnosis. |  |  |
| SCDO:1000018 | treats | A relation between a type of treatment or a specific treatment and a condition (phenotype or disease), where the type of treatment or specific treatment treat the condition. | treated with |  |
| SCDO:0000664 | treated with | A relation between a condition (phenotype or disease) and a type of treatment or a specific treatment, where the condition can be treated with the treatment. | treats |  |
| SCDO:0000475 | uses medical device | A relation between a process (e.g. therapy) and a medical device, where the medical device is used in the process. |  |  |
| SCDO:1110663 | in guideline | A relation between an entity (phenotypic, therapeutic or diagnostic concept) and a guideline or directive. |  |  |
| SCDO:1000059 | has causal molecular phenotype | A relation between a condition (disease such as a Hemoglobinopathy) and a molecular phenotype, where the molecular phenotype is evidence of the condition. |  | has phenotype |
| SCDO:1000222 | causes molecular phenotype | A relation between a genotype and a molecular phenotype, where the genotype has a causal role for the molecular phenotype. |  |  |
| SCDO:1000217 | causes or contributes to molecular phenotype | A relation between an entity (genotype) and a molecular phenotype, where the entity has some causal or contributing role that influences the molecular phenotype. |  |  |
| SCDO:1000218 | has manifestation | A relationship that holds between a condition such as a disease (hemoglobinopathy) and a phenotype (i.e. manifestation), where the phenotype is evidence of the condition. | manifestation of | has phenotype |
| SCDO:1000180 | manifestation of | A relationship that holds between a phenotype (i.e. manifestation) and a condition such as a disease (hemoglobinopathy), wherein the phenotype is eidence of the condition. | has manifestation | phenotype of |
| SCDO:1000202 | caused by | A relationship between a condition (a phenotype or disease) and an entity (e.g. environment) or condition (phenotype or disease) which could be an existing condition or an event) where the entity or second condition has a causal role for the condition being described. |  |  |
| SCDO:0000660 | caused or contributed to by | A relationship between a condition (a phenotype or disease) and an entity (a genotype or genetic variation) where the entity has a causal role for the condition. |  |  |
| SCDO:1000203 | has risk factor | A relation between a condition (disease or phenotype) and an aspect of an individual's life (personal attribute), behavior, an environmental exposure, or an inborn or inherited characteristic that increases the likelihood of the condition. |  |  |
| SCDO:1000204 | risk factor for | A relation between an aspect of an individual's life (personal attribute), behavior, an environmental exposure, or an inborn or inherited characteristic that increases the likelihood of a condition and the condition (disease or phenotype) that it increases the likelihood of. |  |  |
| SCDO:1000199 | induced by | A condition (phenotype or disease) or event (adverse event) induced by an entity (e.g. a drug) or activity (e.g. exercise). |  | caused or contributed to by |
| SCDO:1000172 | has causal genotype | A relation between a condition (hemoglobinopathy) and the genotype that causes the condition. |  | caused or contributed to by |
| SCDO:1000210 | has causal or contributing genetic variation | A relation between a condition (disease or phenotype) or an entity (molecular phenotype) and a genetic variation (e.g. mutation or genetic recombination), where the genetic variation has some causal or contributing role that influences the condition or entity. |  | caused or contributed to by |
| SCDO:1000196 | has contributing genetic variation | A relation between a genotype and a genetic variation (e.g. mutation or genetic recombination), where the genetic variation contributes to the genotype. | contributes to genotype |  |
| SCDO:1000183 | causes or contributes to | A relationship between 'a' and 'b', where 'a' has some causal role for 'b'. |  |  |
| SCDO:1000184 | contributes to genotype | A relationship between an entity (e.g. genetic variation such as mutation or specific allele) and a genotype, where the entity contributes to the genotype. | has contributing genetic variation | causes or contributes to |
| SCDO:1000015 | modifies | A relationship between a disease modifier and a condition (phenotype or disease), entity (aspect of quality of life) or process (therapy), where the disease modifier modifies the condition, entity or process in some way. |  |  |
| SCDO:0000031 | age of onset modified by | A relationship between a phenotype and a disease modifier, where the disease modifier modifies the age of onset of the phenotype. Age of onset is defined as "The age group in which disease manifestations appear." (HPO) |  | modified by |
| SCDO:0000772 | mortality modified by | A relationship between a phenotype and a disease modifier, where the disease modifier modifies the mortality (due to the phenotype) of the SCD sufferer. |  | modified by |
| SCDO:0001139 | temporal pattern modified by | A relationship between a phenotype and a disease modifier, where the disease modifier modifies the temporal pattern of the phenotype. Temporal pattern is defined as "The speed at which disease manifestations appear and develop." (HPO) |  | modified by |
| SCDO:1000005 | modified by | A relationship between a condition (phenotype or disease), entity (aspect of quality of life) or process (therapy) and a disease modifier, where the condition, entity or process is modified by the disease modifier in some way. |  |  |
| SCDO:0000469 | has clinical code | A relationship between a condition (phenotype or disease) and a relevant ICD-10-CM Diagnosis Code. |  |  |
| SCDO:0001297 | has essential measure | A measure that is critical to the collection of the measure at hand or is necessary for the interpretation of results for the measure at hand. Without such information, the data collected would be incomplete or misleading. |  |  |
| SCDO:0001364 | has mode of inheritance | A relation between a particular genotype or disorder and the pattern in which the particular genotype or disorder is passed from one generation to the next. |  |  |
| SCDO:1000188 | studied in | A relation between a condition (Hemoglobinopathy) and a form of research (type of study) that the condition was studied in. |  |  |
| SCDO:1000189 | associated via | A relation between a condition (disease or phenotype) and a type of linking association (e.g. age of onset association). |  |  |

**Section 3: Availability and maintenance**

The SCDO objects are currently in ontology flat file formats in OWL (Ontology Web Language) and OBO (Open Biomedical Ontologies), produced using the ROBOT tool. These files, the ROBOT tool and ontology documentation are available from GitHub (<https://github.com/scdodev/scdo-ontology>). An OBAN (Open Biomedical AssociatioNs) file containing weak or generic disease-phenotype associations supported by an evidence source (provenance) of a class or description of a traceable records, is accessible via a web interface. Issues and new term requests will be tracked via the term tracker and continued updates and maintenance of the SCDO will be performed by the dedicated curation team.

**Section 4: Competency Questions**

Competency questions are rules (axioms or truth, properties and associations) and questions sketched by SCD experts, referred to as competency questions, to check whether the ontology addresses its scope, exploring potential connections/new knowledge. Suggested competency questions related to SCD complications, e.g., acute chest, are shown in **Table S2** and others are categorized below.

**Table S2**: Some of the suggested competency questions associated with SCD complications.

| **Group** | **Hypothesis Generation/Research** | **Knowledge Discovery (known information)** |
| --- | --- | --- |
| 1 - Stroke | Do patients with stroke also have leg ulcer phenotypes (symptoms and signs)?  What is the efficacy of immediate blood transfusion when a patient presents with transient ischemic attack?  Does hypertension contribute to stroke risk in SCD?  Does ethnicity affect the incidence of stroke in adolescent SCD patients?  Does SCD haplotypes increase the risk of stroke? | **SCD** has_phenotype stroke (sign)  Patients with moya moya are at risk for stroke  Fetal Hemoglobin is_modified_by Hydroxyurea which is_preventative_for stroke  Risk of stroke is_modified_by blood transfusion  Anemia is_treated_with blood transfusion which is_preventative_for stroke  Stroke type is_modified_by age  ischemic stroke (ages 2 - 9) older age is a modifier of hemorrhagic (20 -29) stroke  Risk of stroke is _modified_ by education of parents about signs, symptoms and disease modifying therapies |
| 2 - Kidney Disease | Does a severe hemolytic disease increase the risk of acute kidney injury?  Is Hypertension in SCD a risk factor of CKD? SCD patient has _phenotype hypertension has _secondary phenotype of CKD.  What blood pressure in persons with SCD is indicative of “hypertension”. Ie risk/marker of adverse outcomes  What is the best measure of Glomerular filtration rate in SCD?  What are the biomarkers for Kidney dysfunctions?  What the difference between biomarkers and predictors of kidney dysfunctions? | Microalbuminuria increases with age in SCD  Gender  BP  BMI  Creatinemia  APOL1 risk allele  Alpha-thalassemia  HMOX-1 |
| 3 - Pain | Can the severity/ and frequency of acute painful crises be reduced by effective home management techniques  What clinically available biomarkers correlate best with acute painful crisis?  What are the biomarkers for **chronic pain**? | Vaso-occlusive pain in SCD is a common complication but not all patients experience it  Patients who suffer repeated acute painful events may not have typical acute changes associated with pain- tachycardia, hypertension  AVN is a cause of chronic pain in SCD. |
| 4 - Chest Infection | - Are chest infections causes of acute chest syndrome? - What are the common causes of chest infection in SCD? - Are chest infections common in infancy in SCD? - Does asthma increase the risk of a chest infection in SCD? - Do worm infestations increase the risk of asthma in SCD patients? - What is the antibiotic of choice in SCD patients with chest infection? - Do bacterial infections increases pain severity | - Causes of acute chest syndrome include pulmonary embolism, fat embolism, pneumonia, vaso-occlusion - Pneumococcus, mycoplasma, streptococcus, staphylococcus, haemophilus, RSV, HIV, TB, chlamydia are causes of chest infection. - Asthma is more common in patients with SCD than general population - Asthma may predispose to chest infections in SCD due to hypoxemia and inflammation. |
| 5 - Chest | *Does malaria modify ACS?  **What diagnostics distinguish phenotype co-morbid asthma from phenotype RWIS and what is the difference in therapeutics used? | **Persons with SCD who do not have asthma may suffer with recurrent wheezing. |
| 6 - Skin | What are the risk factors for leg ulcer in scd?  What are the genetic modifiers for leg ulcer complication?  What are the effective treatments for leg ulcers  What are the common characteristics of leg ulcers?  Why do some people with SCD not acquire leg ulcer? Are there any predisposing factors?  *How does microbiome impact occurrence or persistence of leg ulcer? | Phenotype has_risk_factor (phenotype, environment, genetic, trauma e.g. hemolysis, low SES, haemoglobin SS)  Phenotype has_genetic_modifier (‘Sequence Feature’ or Genotype)  Phenotype has_treatment Treatment  *Prevalence of leg ulcers varies between geographic locations |

Some other competency questions (CQs) are given below, subdivided into categories: (*1) ‘Discovery’* CQs, which aim at finding all entities of a given type that meet specified criteria; *(2) ‘Descriptive’* CQs which describe attributes of a specific entity input into the query; and (3) *‘Analysis’* CQs ask more open ended research questions that the data may support**.**

**1.** **Discovery CQs**

- Find **methods** used to **diagnose** acute chest syndrome in **adolescent SCD patients.**
- Find phenotypes that the X-ray device can be used to diagnose.
- Find symptoms that characterise central sleep apnea of the nervous system.
- Find distinct SCD phenotypes that result from swelling of the brain.
- Find therapeutic approaches used for the treatment of meningitis in adult SCD patients
- Return all disorders/phenotypes that affect the musculoskeletal system in SCD patients.
- What measures are used to assess the impact of pain crises in adolescent patients?
- What conditions does exercise positively influence in adolescent and adult SCD patients?
- What treatment can be used for end stage renal disease if dialysis is not successful?
- List globin and non-globin genetic loci that have a positive impact on the development of disorders of the urinary system?
- List the causes of acute chest syndrome
- List symptoms that characterize acute chest syndrome?
- What age should we start screening for chronic kidney disease?
- What is the treatment of pulmonary hypertension?
- Is Hypertension a risk factor for stroke?
- What are the different types of stroke (transit in )?
- Are there Genome Wide association studies of Sickle Cell Disease
- What are the consequences of stroke?
- What is the blood pressure for patients of sickle cell?
- What are oximetry measures normally found in SCD?
- What are the pre-natal screening tests for Sickle Cell Disease? There was no description in the ontology of the tests apart from the curator notes were also not helpful?
- Do these genetic modifiers affect Sickle Cell Disease?
- Which instrument to use when?
- What is the threshold of fetal haemoglobin affect disease?
- How is bone pain treated?
- How is Ataxia diagnosed?
- Can Meningitis be treated with antibiotics?
- Which age group is most affected by meningitis?​
- How is pain classified in scd? This question cannot be answered by the SCDO.
- Is there a scale of severity for pain in SCD? (This might be more of a website content question)
- Is there any global scale of severity for SCD?. This question cannot be answered by the SCDO
- What are the different causes/risk factors for enlarged spleen in children with SCD?
- Is it necessary to maintain children under prophylaxis after splenectomy? For how much time?
- Is there need for pneumococcal vaccination beyond 5 years of age?
- What should be the first line antibiotics for an under five SCD child presenting with fever above 39°
- What are the different clinical/radiologic features of stroke in SCD?
- How does pain affect the quality of life in people with sickle cell disease?
- How does socio-economic status impact disease severity
- What are the different environmental factors that influence the occurrence of a stroke episode?
- What is the evidence in relation to social determinants as they impact outcomes in sickle cell disease
- When does hydroxyurea start in SCD? This question cannot be answered by the SCDO
- How does the hospital environment affect young people with SCD ability to respond positively with treatment regimen?
- How to address toxicity of hydroxyurea in SB0 patients with splenomegaly?
- What is the role of nutrition/specific nutrients in improving health outcomes of SCD patients?
- How stigma influence health outcomes/ experiences of SCD patients?
- How do I screen for sleep disordered breathing in SCD?
- How does stress affect the experience of pain?
- Is there indication for HSCT for all patients that have compatible donors?
- How does kidney disease affect anemia in SCD?
- How do I screen for depression and beginning at what age?

**2. ‘Descriptive’ CQs**

- What is the BAI (Beck Anxiety Inventory)? What is it used for?
- What SCD phenotypes do viral agents cause?
- What is hypertension in Sickle Cell Disease how is that different from other hypertension?
- What is acute stroke, what is chronic stroke?
- What is the role of a scan? Imagining Doppler scan? T
- What is the role of a transplant in the treatment of stroke?
- What is a crisis?
- What are vaso-occlusive crisis? This question is answered by the SCDO but it is incomplete?
- Describe iron overload – is it a (diagnosis)
- How does the healthcare facility modify Sickle Cell?
- How do Gene/Loci/ CNVs etc modify Sickle Cell?
- What symptoms characterise Pulmonary hypertension
- How are Sickle Cell Patients Rehabilitated after a stroke?
- What is the role of echo in diagnosis of Sickle Cell Disease?
- Describe the different way of diagnosing Sickle Cell Disease
- What would be the best method for diagnosing Sickle Cell Disease
- What is the role of spectroscopy in newborn Screening of Sickle Cell.
- Describe the indications of transfusion in Acute Chest Syndrome.
- Describe the mortality due to ACS in SCD.
- What are the complications of ACS in SCD?

**3.** **‘Analysis’ CQs**

- When do you transplant for kidney disease?
- Can you cure kidney disease?
- How do you prevent kidney disease?
- What are the cardiovascular questions in kidney disease?
- What are the risk factor for sickle cell?
- Are there genetic modifiers of Sickle Cell Disease?
- When does one decide on abortion?
- Are there modifiers which can be engineered into therapeutics eg built into signalling transduction?
- What are the follow-up studies after diagnosis?
